# Methyl 4-(4-methoxy­phen­yl)-1,2,3,3a,4,4a,5,12c-octa­hydro­benzo[*f*]chromeno[3,4-*b*]pyrrolizine-4a-carboxyl­ate

**DOI:** 10.1107/S1600536809027639

**Published:** 2009-07-22

**Authors:** S. Nirmala, E. Theboral Sugi Kamala, L. Sudha, S. Kathiravan, R. Raghunathan

**Affiliations:** a Department of Physics, Easwari Engineering College, Ramapuram, Chennai 600 089, India; b Department of Physics, SRM University, Ramapuram Campus, Chennai 600 089, India; c Department of Organic Chemistry, University of Madras, Guindy Campus, Chennai 600 025, India

## Abstract

In the title compound, C_27_H_27_NO_4_, both the pyrrolidine rings in the pyrrolizine ring system adopt envelope conformations, whereas the dihydro­pyran ring adopts a half-chair conformation. The methoxy­phenyl group is oriented at an angle of 53.72 (4)° with respect to the naphthalene ring system. Intra­molecular C—H⋯O hydrogen bonds are observed. The crystal structure is stabilized by weak inter­molecular C—H⋯π inter­actions.

## Related literature

For the biological activity of pyrrolizine derivatives, see: Amal Raj *et al.* (2003 ▶); Atal (1978 ▶); Denny (2001 ▶); Suzuki *et al.* (1994 ▶). For a related structure, see: Ramesh *et al.* (2007 ▶). For ring-puckering parameters, see: Cremer & Pople (1975 ▶).
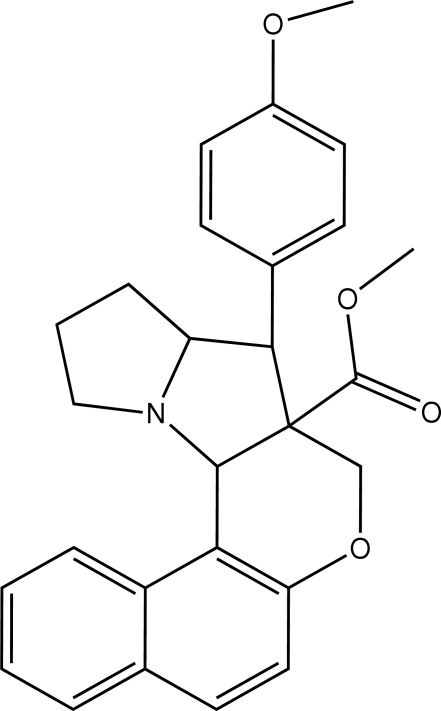


## Experimental

### 

#### Crystal data

C_27_H_27_NO_4_
*M* *_r_* = 429.50
Triclinic, 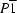

*a* = 8.7484 (4) Å
*b* = 11.4284 (5) Å
*c* = 11.4444 (6) Å
α = 104.127 (2)°
β = 91.824 (3)°
γ = 101.555 (2)°
*V* = 1083.19 (9) Å^3^
*Z* = 2
Mo *K*α radiation
μ = 0.09 mm^−1^
*T* = 293 K
0.30 × 0.25 × 0.20 mm

#### Data collection

Bruker Kappa APEXII area-detector diffractometer
Absorption correction: multi-scan (Blessing, 1995 ▶) *T*_min_ = 0.974, *T*_max_ = 0.983
22194 measured reflections
4310 independent reflections
2976 reflections with *I* > 2σ(*I*)
*R*_int_ = 0.029

#### Refinement

*R*[*F* ^2^ > 2σ(*F* ^2^)] = 0.046
*wR*(*F* ^2^) = 0.131
*S* = 1.03
4310 reflections
289 parameters
H-atom parameters constrained
Δρ_max_ = 0.35 e Å^−3^
Δρ_min_ = −0.16 e Å^−3^

### 

Data collection: *APEX2* (Bruker, 2004 ▶); cell refinement: *SAINT* (Bruker, 2004 ▶); data reduction: *SAINT*; program(s) used to solve structure: *SHELXS97* (Sheldrick, 2008 ▶); program(s) used to refine structure: *SHELXL97* (Sheldrick, 2008 ▶); molecular graphics: *ORTEP-3* (Farrugia, 1997 ▶); software used to prepare material for publication: *PLATON* (Spek, 2009 ▶).

## Supplementary Material

Crystal structure: contains datablocks I, global. DOI: 10.1107/S1600536809027639/bt5001sup1.cif

Structure factors: contains datablocks I. DOI: 10.1107/S1600536809027639/bt5001Isup2.hkl

Additional supplementary materials:  crystallographic information; 3D view; checkCIF report

## Figures and Tables

**Table 1:** Hydrogen-bond geometry (Å, °)

*D*—H⋯*A*	*D*—H	H⋯*A*	*D*⋯*A*	*D*—H⋯*A*
C1—H1⋯O2	0.98	2.32	2.792 (2)	108
C23—H23⋯*Cg*1^i^	0.93	2.92	3.633 (3)	135
C25—H25*C*⋯*Cg*2^ii^	0.96	2.90	3.715 (3)	143

Symmetry codes: (i) 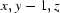; (ii) 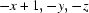. *Cg1* is the centroid of the C3–C8 ring and *Cg2* is the centroid of the C19–C24 ring.

**Fig. 1.**
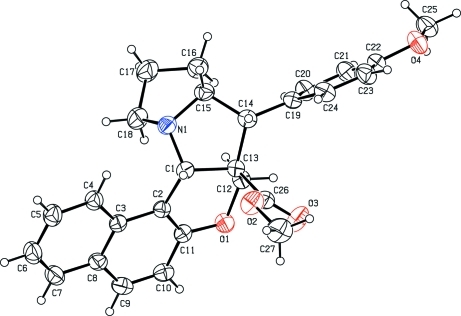
The molecular structure of the title compound, with 30% probability displacement ellipsoids.

## supplementary crystallographic information

### Comment

Pyrrolizidine alkaloids occur in more than 40 genera, and are responsible for heavy losses of livestock and poisoning in man due to their hepatotoxity. These alkaloids are also reported to possess a number of other biological activities (Atal, 1978) and are used as DNA minor groove alkylating agents (Denny, 2001). Substituted pyrrolidines have gained much importance because they are the structural elements of many alkaloids. It has been found that they exhibit antifungal activity against various pathogens (Amal Raj *et al.*, 2003). Optically active pyrrolidine derivatives have been used as intermediates in controlled asymmetric synthesis (Suzuki *et al.*, 1994). In view of its biological importance, the crystal structure determination of the title compound was undertaken.

A displacement ellipsoid plot of the title compound is shown in Fig. 1. The pyrrolizine ring system is folded about the bridging N1—C15 bond, as observed in related structures (Ramesh *et al.*, 2007). The sum of bond angles around atom N1 [337.9 (7)°] is in accordance with *sp*^3^ hybridization. The napthalene ring system C2-C11 and the methoxyphenyl group C19—C25/O4 are oriented at an angle of 53.72 (4)° with respect to each other. The methoxy group is almost coplanar with the C19—C24 benzene ring [C25—O4—C22—C23 = -174.4 (2)°]. The heterocyclic ring O1/C1/C2/C11—C13 of the chromenopyrrolizine unit adopt  a half chair conformation with puckering parameters *q_2 _*= 0.381 (2) Å, *q_3 _*= -0.281 (2) Å and *φ*= -91.3 (3)° (Cremer and Pople, 1975). In the pyrrolizine ring system, both the pyrrolidine rings, N1/C1/C13—C15 and N1/C15—C18 adopt  envelope conformation with puckering parameters *q_2 _*= 0.352 (2) Å, *φ*= 78.4 (3)° and *q_2_* = 0.402 (3) Å, *φ*= 100.9 (3)° (Cremer and Pople, 1975) respectively. In the ring N1/C1/C13—C15, atom C13 deviates by 0.547 (8) Å from the least-square plane through the remaining four atoms, whereas in the ring N1/C15—C18, atom C17 deviates by -0.616 (4) Å from the least-squares plane through the remaining four atoms.

The crystal packing is stabilized by intramolecular C—H···O and weak intermolecular C—H···π (C23—H23···*Cg1*; *Cg1* is the centroid of the C3—C8 ring and C25—H25C···*Cg2*; *Cg2* is the centroid of the C19—C24 ring) interactions (Table 1).

### Experimental

A mixture of (*Z*)-methyl-2-((1-formylnaphthalen-2-yloxy)methyl)-3-(4-methoxyphenyl) acrylate (20 mmol) and proline (30 mmol) were refluxed in benzene for 20 h and the solvent was removed under reduced pressure. The crude product was subjected to column chromatography to get the pure product. A chloroform and methanol (1:1) solvent mixture was used for the crystallization using the slow evaporation method.

### Refinement

H atoms were placed in idealized positions and allowed to ride on their parent atoms, with C—H = 0.93, 0.98, 0.97 and 0.96 Å for aromatic, methine, methylene and methyl H respectively, and *U*iso(H) = 1.5U_eq_(C) for methyl H and *U*iso(H) = 1.2U_eq_(C) for all other H atoms.

### Crystal data

**Table:**

C_27_H_27_NO_4_	*Z* = 2
*M**_r_* = 429.50	*F*(000) = 456
Triclinic, *P*1	*D*_x_ = 1.317 Mg m^−^^3^
Hall symbol: -P 1	Mo *K*α radiation, λ = 0.71073 Å
*a* = 8.7484 (4) Å	Cell parameters from 6981 reflections
*b* = 11.4284 (5) Å	θ = 2.3–26.0°
*c* = 11.4444 (6) Å	µ = 0.09 mm^−^^1^
α = 104.127 (2)°	*T* = 293 K
β = 91.824 (3)°	Prism, yellow
γ = 101.555 (2)°	0.30 × 0.25 × 0.20 mm
*V* = 1083.19 (9) Å^3^	

### Data collection

**Table:**

Bruker Kappa APEXII area-detector diffractometer	4310 independent reflections
Radiation source: fine-focus sealed tube	2976 reflections with *I* > 2σ(*I*)
graphite	*R*_int_ = 0.029
ω and φ scans	θ_max_ = 26.2°, θ_min_ = 2.3°
Absorption correction: multi-scan (Blessing, 1995)	*h* = −10→10
*T*_min_ = 0.974, *T*_max_ = 0.983	*k* = −14→14
22194 measured reflections	*l* = −14→14

### Refinement

**Table:**

Refinement on *F*^2^	Primary atom site location: structure-invariant direct methods
Least-squares matrix: full	Secondary atom site location: difference Fourier map
*R*[*F*^2^ > 2σ(*F*^2^)] = 0.046	Hydrogen site location: inferred from neighbouring sites
*wR*(*F*^2^) = 0.131	H-atom parameters constrained
*S* = 1.03	*w* = 1/[σ^2^(*F*_o_^2^) + (0.0551*P*)^2^ + 0.3082*P*] where *P* = (*F*_o_^2^ + 2*F*_c_^2^)/3
4310 reflections	(Δ/σ)_max_ < 0.001
289 parameters	Δρ_max_ = 0.35 e Å^−^^3^
0 restraints	Δρ_min_ = −0.16 e Å^−^^3^

### Special details

**Table:**

Geometry. All e.s.d.'s (except the e.s.d. in the dihedral angle between two l.s. planes) are estimated using the full covariance matrix. The cell e.s.d.'s are taken into account individually in the estimation of e.s.d.'s in distances, angles and torsion angles; correlations between e.s.d.'s in cell parameters are only used when they are defined by crystal symmetry. An approximate (isotropic) treatment of cell e.s.d.'s is used for estimating e.s.d.'s involving l.s. planes.
Refinement. Refinement of *F*^2^ against ALL reflections. The weighted *R*-factor *wR* and goodness of fit *S* are based on *F*^2^, conventional *R*-factors *R* are based on *F*, with *F* set to zero for negative *F*^2^. The threshold expression of *F*^2^ > σ(*F*^2^) is used only for calculating *R*-factors(gt) *etc*. and is not relevant to the choice of reflections for refinement. *R*-factors based on *F*^2^ are statistically about twice as large as those based on *F*, and *R*- factors based on ALL data will be even larger.

### Fractional atomic coordinates and isotropic or equivalent isotropic displacement parameters (Å^2^)

**Table:**

	*x*	*y*	*z*	*U*_iso_*/*U*_eq_	
C1	0.3428 (2)	0.57705 (16)	0.31111 (15)	0.0445 (4)	
H1	0.2866	0.5752	0.3836	0.053*	
C2	0.26413 (19)	0.64642 (15)	0.23992 (15)	0.0431 (4)	
C3	0.2290 (2)	0.76283 (16)	0.29772 (16)	0.0464 (4)	
C4	0.2730 (2)	0.82056 (17)	0.42105 (17)	0.0548 (5)	
H4	0.3284	0.7834	0.4669	0.066*	
C5	0.2352 (3)	0.93069 (19)	0.4741 (2)	0.0657 (6)	
H5	0.2634	0.9668	0.5559	0.079*	
C6	0.1550 (3)	0.9895 (2)	0.4072 (2)	0.0716 (6)	
H6	0.1308	1.0648	0.4441	0.086*	
C7	0.1123 (2)	0.93691 (19)	0.2880 (2)	0.0661 (6)	
H7	0.0594	0.9770	0.2437	0.079*	
C8	0.1466 (2)	0.82244 (17)	0.23017 (18)	0.0521 (5)	
C9	0.0967 (2)	0.76322 (19)	0.10731 (19)	0.0582 (5)	
H9	0.0404	0.8008	0.0627	0.070*	
C10	0.1295 (2)	0.65353 (18)	0.05396 (17)	0.0538 (5)	
H10	0.0945	0.6155	−0.0266	0.065*	
C11	0.2164 (2)	0.59630 (16)	0.11972 (16)	0.0462 (4)	
C12	0.3576 (2)	0.43780 (17)	0.10894 (15)	0.0479 (4)	
H12A	0.4615	0.4851	0.1043	0.057*	
H12B	0.3522	0.3530	0.0643	0.057*	
C13	0.3321 (2)	0.44172 (15)	0.23988 (14)	0.0425 (4)	
C14	0.4659 (2)	0.40757 (17)	0.30803 (15)	0.0477 (4)	
H14	0.4235	0.3968	0.3839	0.057*	
C15	0.5928 (2)	0.52759 (18)	0.34653 (17)	0.0546 (5)	
H15	0.6344	0.5349	0.4291	0.066*	
C16	0.7312 (2)	0.5558 (2)	0.2738 (2)	0.0688 (6)	
H16A	0.8211	0.5289	0.3014	0.083*	
H16B	0.7050	0.5168	0.1882	0.083*	
C17	0.7619 (3)	0.6949 (2)	0.2989 (3)	0.0855 (8)	
H17A	0.8176	0.7342	0.3778	0.103*	
H17B	0.8215	0.7239	0.2376	0.103*	
C18	0.5994 (3)	0.7186 (2)	0.2943 (3)	0.0773 (7)	
H18A	0.5985	0.8025	0.3386	0.093*	
H18B	0.5579	0.7056	0.2114	0.093*	
C19	0.5143 (2)	0.28744 (16)	0.25383 (15)	0.0454 (4)	
C20	0.5936 (2)	0.26369 (18)	0.15102 (17)	0.0542 (5)	
H20	0.6166	0.3239	0.1085	0.065*	
C21	0.6397 (2)	0.15321 (18)	0.10964 (18)	0.0570 (5)	
H21	0.6940	0.1404	0.0407	0.068*	
C22	0.6059 (2)	0.06275 (17)	0.16960 (19)	0.0562 (5)	
C23	0.5197 (3)	0.0811 (2)	0.2683 (2)	0.0646 (6)	
H23	0.4905	0.0184	0.3071	0.077*	
C24	0.4769 (2)	0.19191 (19)	0.30954 (18)	0.0570 (5)	
H24	0.4206	0.2034	0.3775	0.068*	
C25	0.7526 (3)	−0.0636 (2)	0.0442 (2)	0.0881 (8)	
H25A	0.7767	−0.1440	0.0309	0.132*	
H25B	0.8476	−0.0015	0.0663	0.132*	
H25C	0.7019	−0.0565	−0.0286	0.132*	
C26	0.1780 (2)	0.35504 (17)	0.24530 (17)	0.0488 (4)	
C27	−0.0206 (3)	0.3004 (3)	0.3694 (3)	0.0902 (8)	
H27A	−0.0501	0.3283	0.4498	0.135*	
H27B	−0.0039	0.2179	0.3572	0.135*	
H27C	−0.1026	0.3010	0.3119	0.135*	
N1	0.50894 (18)	0.62899 (14)	0.35141 (14)	0.0554 (4)	
O1	0.24421 (15)	0.48642 (12)	0.05440 (10)	0.0547 (3)	
O2	0.12217 (17)	0.38163 (14)	0.35295 (13)	0.0697 (4)	
O3	0.11479 (19)	0.27038 (15)	0.16475 (14)	0.0831 (5)	
O4	0.6519 (2)	−0.04732 (14)	0.13839 (16)	0.0803 (5)	

### Atomic displacement parameters (Å^2^)

**Table:**

	*U*^11^	*U*^22^	*U*^33^	*U*^12^	*U*^13^	*U*^23^
C1	0.0434 (10)	0.0490 (10)	0.0396 (9)	0.0085 (8)	0.0057 (7)	0.0093 (8)
C2	0.0397 (10)	0.0457 (10)	0.0450 (9)	0.0063 (7)	0.0072 (7)	0.0155 (8)
C3	0.0380 (10)	0.0458 (10)	0.0550 (11)	0.0044 (7)	0.0110 (8)	0.0152 (8)
C4	0.0537 (12)	0.0510 (11)	0.0574 (11)	0.0113 (9)	0.0083 (9)	0.0091 (9)
C5	0.0664 (14)	0.0559 (12)	0.0688 (13)	0.0131 (10)	0.0135 (11)	0.0036 (10)
C6	0.0648 (14)	0.0525 (12)	0.0970 (18)	0.0187 (10)	0.0201 (12)	0.0112 (12)
C7	0.0532 (13)	0.0577 (12)	0.0948 (17)	0.0188 (10)	0.0131 (11)	0.0267 (12)
C8	0.0374 (10)	0.0532 (11)	0.0698 (13)	0.0089 (8)	0.0118 (9)	0.0232 (10)
C9	0.0466 (11)	0.0692 (13)	0.0683 (13)	0.0148 (9)	0.0059 (9)	0.0331 (11)
C10	0.0493 (11)	0.0649 (12)	0.0495 (10)	0.0084 (9)	0.0026 (8)	0.0223 (9)
C11	0.0451 (10)	0.0483 (10)	0.0458 (10)	0.0061 (8)	0.0076 (8)	0.0160 (8)
C12	0.0557 (11)	0.0494 (10)	0.0404 (9)	0.0138 (8)	0.0082 (8)	0.0124 (8)
C13	0.0447 (10)	0.0469 (10)	0.0368 (9)	0.0104 (8)	0.0062 (7)	0.0117 (7)
C14	0.0511 (11)	0.0567 (11)	0.0381 (9)	0.0156 (8)	0.0078 (8)	0.0135 (8)
C15	0.0521 (11)	0.0636 (12)	0.0447 (10)	0.0167 (9)	−0.0008 (8)	0.0046 (9)
C16	0.0547 (13)	0.0684 (14)	0.0753 (14)	0.0125 (10)	0.0114 (10)	0.0036 (11)
C17	0.0533 (14)	0.0721 (15)	0.118 (2)	−0.0002 (11)	0.0134 (13)	0.0102 (14)
C18	0.0553 (14)	0.0623 (13)	0.1104 (19)	0.0004 (10)	0.0123 (12)	0.0241 (13)
C19	0.0459 (10)	0.0520 (10)	0.0412 (9)	0.0129 (8)	0.0048 (8)	0.0154 (8)
C20	0.0625 (12)	0.0548 (11)	0.0531 (11)	0.0183 (9)	0.0173 (9)	0.0220 (9)
C21	0.0614 (13)	0.0594 (12)	0.0546 (11)	0.0225 (10)	0.0146 (9)	0.0140 (10)
C22	0.0542 (12)	0.0509 (11)	0.0652 (12)	0.0184 (9)	−0.0026 (10)	0.0135 (10)
C23	0.0704 (14)	0.0618 (13)	0.0744 (14)	0.0199 (11)	0.0102 (11)	0.0362 (11)
C24	0.0590 (12)	0.0685 (13)	0.0535 (11)	0.0206 (10)	0.0137 (9)	0.0273 (10)
C25	0.0778 (17)	0.0780 (16)	0.105 (2)	0.0431 (13)	−0.0010 (14)	−0.0032 (14)
C26	0.0499 (11)	0.0510 (11)	0.0488 (10)	0.0124 (9)	0.0060 (8)	0.0175 (9)
C27	0.0667 (16)	0.1030 (19)	0.111 (2)	0.0060 (14)	0.0337 (14)	0.0537 (17)
N1	0.0495 (9)	0.0541 (9)	0.0567 (9)	0.0097 (7)	0.0010 (7)	0.0046 (8)
O1	0.0679 (9)	0.0583 (8)	0.0383 (6)	0.0175 (6)	−0.0014 (6)	0.0105 (6)
O2	0.0631 (9)	0.0799 (10)	0.0636 (9)	0.0022 (7)	0.0239 (7)	0.0222 (8)
O3	0.0781 (11)	0.0744 (10)	0.0743 (10)	−0.0156 (8)	0.0073 (8)	0.0027 (9)
O4	0.0883 (12)	0.0606 (9)	0.1009 (12)	0.0360 (8)	0.0093 (9)	0.0210 (9)

### Geometric parameters (Å, °)

**Table:**

C1—N1	1.468 (2)	C15—C16	1.517 (3)
C1—C2	1.505 (2)	C15—H15	0.9800
C1—C13	1.545 (2)	C16—C17	1.511 (3)
C1—H1	0.9800	C16—H16A	0.9700
C2—C11	1.370 (2)	C16—H16B	0.9700
C2—C3	1.432 (2)	C17—C18	1.501 (3)
C3—C4	1.409 (3)	C17—H17A	0.9700
C3—C8	1.414 (3)	C17—H17B	0.9700
C4—C5	1.367 (3)	C18—N1	1.462 (3)
C4—H4	0.9300	C18—H18A	0.9700
C5—C6	1.391 (3)	C18—H18B	0.9700
C5—H5	0.9300	C19—C20	1.383 (2)
C6—C7	1.355 (3)	C19—C24	1.384 (3)
C6—H6	0.9300	C20—C21	1.380 (2)
C7—C8	1.410 (3)	C20—H20	0.9300
C7—H7	0.9300	C21—C22	1.365 (3)
C8—C9	1.415 (3)	C21—H21	0.9300
C9—C10	1.344 (3)	C22—O4	1.366 (2)
C9—H9	0.9300	C22—C23	1.375 (3)
C10—C11	1.405 (3)	C23—C24	1.369 (3)
C10—H10	0.9300	C23—H23	0.9300
C11—O1	1.368 (2)	C24—H24	0.9300
C12—O1	1.426 (2)	C25—O4	1.415 (3)
C12—C13	1.513 (2)	C25—H25A	0.9600
C12—H12A	0.9700	C25—H25B	0.9600
C12—H12B	0.9700	C25—H25C	0.9600
C13—C26	1.518 (3)	C26—O3	1.187 (2)
C13—C14	1.550 (2)	C26—O2	1.328 (2)
C14—C19	1.511 (2)	C27—O2	1.447 (3)
C14—C15	1.540 (3)	C27—H27A	0.9600
C14—H14	0.9800	C27—H27B	0.9600
C15—N1	1.482 (2)	C27—H27C	0.9600
			
N1—C1—C2	116.27 (15)	C14—C15—H15	107.5
N1—C1—C13	106.30 (14)	C17—C16—C15	102.37 (17)
C2—C1—C13	111.88 (14)	C17—C16—H16A	111.3
N1—C1—H1	107.3	C15—C16—H16A	111.3
C2—C1—H1	107.3	C17—C16—H16B	111.3
C13—C1—H1	107.3	C15—C16—H16B	111.3
C11—C2—C3	118.40 (16)	H16A—C16—H16B	109.2
C11—C2—C1	120.71 (15)	C18—C17—C16	102.44 (18)
C3—C2—C1	120.79 (15)	C18—C17—H17A	111.3
C4—C3—C8	118.32 (17)	C16—C17—H17A	111.3
C4—C3—C2	122.10 (17)	C18—C17—H17B	111.3
C8—C3—C2	119.58 (17)	C16—C17—H17B	111.3
C5—C4—C3	120.65 (19)	H17A—C17—H17B	109.2
C5—C4—H4	119.7	N1—C18—C17	104.1 (2)
C3—C4—H4	119.7	N1—C18—H18A	110.9
C4—C5—C6	120.9 (2)	C17—C18—H18A	110.9
C4—C5—H5	119.6	N1—C18—H18B	110.9
C6—C5—H5	119.6	C17—C18—H18B	110.9
C7—C6—C5	119.9 (2)	H18A—C18—H18B	109.0
C7—C6—H6	120.1	C20—C19—C24	116.18 (17)
C5—C6—H6	120.1	C20—C19—C14	125.22 (16)
C6—C7—C8	121.1 (2)	C24—C19—C14	118.60 (15)
C6—C7—H7	119.4	C21—C20—C19	121.92 (18)
C8—C7—H7	119.4	C21—C20—H20	119.0
C7—C8—C3	119.12 (19)	C19—C20—H20	119.0
C7—C8—C9	122.01 (19)	C22—C21—C20	120.22 (18)
C3—C8—C9	118.83 (17)	C22—C21—H21	119.9
C10—C9—C8	121.16 (18)	C20—C21—H21	119.9
C10—C9—H9	119.4	C21—C22—O4	124.41 (19)
C8—C9—H9	119.4	C21—C22—C23	119.14 (18)
C9—C10—C11	120.08 (18)	O4—C22—C23	116.45 (19)
C9—C10—H10	120.0	C24—C23—C22	119.95 (19)
C11—C10—H10	120.0	C24—C23—H23	120.0
O1—C11—C2	123.82 (16)	C22—C23—H23	120.0
O1—C11—C10	114.28 (15)	C23—C24—C19	122.43 (18)
C2—C11—C10	121.87 (17)	C23—C24—H24	118.8
O1—C12—C13	112.03 (14)	C19—C24—H24	118.8
O1—C12—H12A	109.2	O4—C25—H25A	109.5
C13—C12—H12A	109.2	O4—C25—H25B	109.5
O1—C12—H12B	109.2	H25A—C25—H25B	109.5
C13—C12—H12B	109.2	O4—C25—H25C	109.5
H12A—C12—H12B	107.9	H25A—C25—H25C	109.5
C12—C13—C26	108.88 (15)	H25B—C25—H25C	109.5
C12—C13—C1	109.10 (14)	O3—C26—O2	122.82 (18)
C26—C13—C1	114.22 (14)	O3—C26—C13	124.82 (17)
C12—C13—C14	113.80 (14)	O2—C26—C13	112.33 (16)
C26—C13—C14	109.63 (14)	O2—C27—H27A	109.5
C1—C13—C14	101.16 (13)	O2—C27—H27B	109.5
C19—C14—C15	118.94 (15)	H27A—C27—H27B	109.5
C19—C14—C13	118.49 (14)	O2—C27—H27C	109.5
C15—C14—C13	104.79 (14)	H27A—C27—H27C	109.5
C19—C14—H14	104.3	H27B—C27—H27C	109.5
C15—C14—H14	104.3	C18—N1—C1	119.67 (16)
C13—C14—H14	104.3	C18—N1—C15	108.60 (15)
N1—C15—C16	105.10 (16)	C1—N1—C15	109.70 (14)
N1—C15—C14	105.46 (14)	C11—O1—C12	116.10 (13)
C16—C15—C14	122.97 (16)	C26—O2—C27	116.63 (18)
N1—C15—H15	107.5	C22—O4—C25	117.36 (18)
C16—C15—H15	107.5		
			
N1—C1—C2—C11	111.83 (18)	C13—C14—C15—N1	24.27 (17)
C13—C1—C2—C11	−10.5 (2)	C19—C14—C15—C16	39.4 (3)
N1—C1—C2—C3	−72.0 (2)	C13—C14—C15—C16	−95.8 (2)
C13—C1—C2—C3	165.67 (14)	N1—C15—C16—C17	28.2 (2)
C11—C2—C3—C4	179.67 (17)	C14—C15—C16—C17	148.5 (2)
C1—C2—C3—C4	3.4 (3)	C15—C16—C17—C18	−40.8 (2)
C11—C2—C3—C8	0.1 (2)	C16—C17—C18—N1	38.3 (3)
C1—C2—C3—C8	−176.21 (16)	C15—C14—C19—C20	−58.3 (2)
C8—C3—C4—C5	0.8 (3)	C13—C14—C19—C20	70.9 (2)
C2—C3—C4—C5	−178.80 (17)	C15—C14—C19—C24	121.98 (19)
C3—C4—C5—C6	−1.2 (3)	C13—C14—C19—C24	−108.8 (2)
C4—C5—C6—C7	0.6 (3)	C24—C19—C20—C21	−3.1 (3)
C5—C6—C7—C8	0.5 (3)	C14—C19—C20—C21	177.25 (18)
C6—C7—C8—C3	−0.9 (3)	C19—C20—C21—C22	0.7 (3)
C6—C7—C8—C9	177.03 (19)	C20—C21—C22—O4	−177.28 (18)
C4—C3—C8—C7	0.2 (3)	C20—C21—C22—C23	2.8 (3)
C2—C3—C8—C7	179.84 (16)	C21—C22—C23—C24	−3.8 (3)
C4—C3—C8—C9	−177.72 (17)	O4—C22—C23—C24	176.24 (19)
C2—C3—C8—C9	1.9 (2)	C22—C23—C24—C19	1.4 (3)
C7—C8—C9—C10	−179.41 (18)	C20—C19—C24—C23	2.0 (3)
C3—C8—C9—C10	−1.5 (3)	C14—C19—C24—C23	−178.29 (18)
C8—C9—C10—C11	−0.9 (3)	C12—C13—C26—O3	22.6 (3)
C3—C2—C11—O1	179.38 (15)	C1—C13—C26—O3	144.8 (2)
C1—C2—C11—O1	−4.3 (3)	C14—C13—C26—O3	−102.5 (2)
C3—C2—C11—C10	−2.5 (3)	C12—C13—C26—O2	−159.46 (15)
C1—C2—C11—C10	173.79 (16)	C1—C13—C26—O2	−37.2 (2)
C9—C10—C11—O1	−178.78 (16)	C14—C13—C26—O2	75.45 (18)
C9—C10—C11—C2	3.0 (3)	C17—C18—N1—C1	−147.84 (18)
O1—C12—C13—C26	65.72 (19)	C17—C18—N1—C15	−20.9 (2)
O1—C12—C13—C1	−59.54 (19)	C2—C1—N1—C18	−17.6 (2)
O1—C12—C13—C14	−171.67 (14)	C13—C1—N1—C18	107.66 (18)
N1—C1—C13—C12	−87.59 (17)	C2—C1—N1—C15	−144.03 (15)
C2—C1—C13—C12	40.32 (19)	C13—C1—N1—C15	−18.77 (18)
N1—C1—C13—C26	150.32 (15)	C16—C15—N1—C18	−4.7 (2)
C2—C1—C13—C26	−81.77 (18)	C14—C15—N1—C18	−136.01 (17)
N1—C1—C13—C14	32.66 (17)	C16—C15—N1—C1	127.72 (16)
C2—C1—C13—C14	160.56 (14)	C14—C15—N1—C1	−3.54 (19)
C12—C13—C14—C19	−53.0 (2)	C2—C11—O1—C12	−14.2 (2)
C26—C13—C14—C19	69.19 (19)	C10—C11—O1—C12	167.62 (15)
C1—C13—C14—C19	−169.85 (14)	C13—C12—O1—C11	46.8 (2)
C12—C13—C14—C15	82.45 (17)	O3—C26—O2—C27	1.0 (3)
C26—C13—C14—C15	−155.35 (14)	C13—C26—O2—C27	−176.96 (17)
C1—C13—C14—C15	−34.40 (16)	C21—C22—O4—C25	5.7 (3)
C19—C14—C15—N1	159.48 (15)	C23—C22—O4—C25	−174.4 (2)

### Hydrogen-bond geometry (Å, °)

**Table:**

*D*—H···*A*	*D*—H	H···*A*	*D*···*A*	*D*—H···*A*
C1—H1···O2	0.98	2.32	2.792 (2)	108
C23—H23···Cg1^i^	0.93	2.92	3.633 (3)	135
C25—H25C···Cg2^ii^	0.96	2.90	3.715 (3)	143

Symmetry codes: (i) *x*, *y*−1, *z*; (ii) −*x*+1, −*y*, −*z*.
